# A stitched jaw in the newborn: subglosso-palatal membrane

**DOI:** 10.11604/pamj.2018.31.80.16591

**Published:** 2018-10-03

**Authors:** Pelin Dogan, Ipek Güney Varal

**Affiliations:** 1 Department of Pediatrics, Division of Neonatology, University of Health Sciences, Bursa Yüksek Ihtisas Teaching Hospital, Bursa, Turkey

**Keywords:** Subglosso-palatal membrane, congenital anomalies, infant

## Image in medicine

Subglosso-palatal membrane (SPM) is an extremely rare entity and only a few neonatal cases have been reported in the literature. Anatomically, SPM is a fibrous tissue, extending from the floor of the mouth to the palate. Majority of the cases are recognized during the first feeding with the inability of opening the mouth and latching, thus feeding difficulty is the major sign that prompt the physician to consider the diagnosis. Simple surgical resection of the fibrous band is sufficient for the treatment; however, the practical importance of this entity is that physicians should keep in mind the possible co-occurrence of other congenital anomalies such as cleft lip and palate, micrognathia, microglossia and temporomandibular joint disorders in these infants. We present the case of a 3230g male infant was born at 40 weeks gestation to a 30-year-old gravida 2 para 1 mother via cesarean section. Upon delivery he was noted to have severely restricted oral opening due to a mucous intra-oral band vertically connecting the floor of the mouth to the midline of the hard palate. Tongue movements were intact. The pregnancy history was unremarkable, the mother denied having taken any drugs, there was no family history of congenital anomalies or consanguinity. Immediate surgical resection of the aberrant tissue was performed in the operating room with minimal hemorrhage following excision. The physical examination of the infant was otherwise unremarkable and detailed genetic assessment was also normal. The infant was discharged home on full oral feedings on day 5.

**Figure 1:**
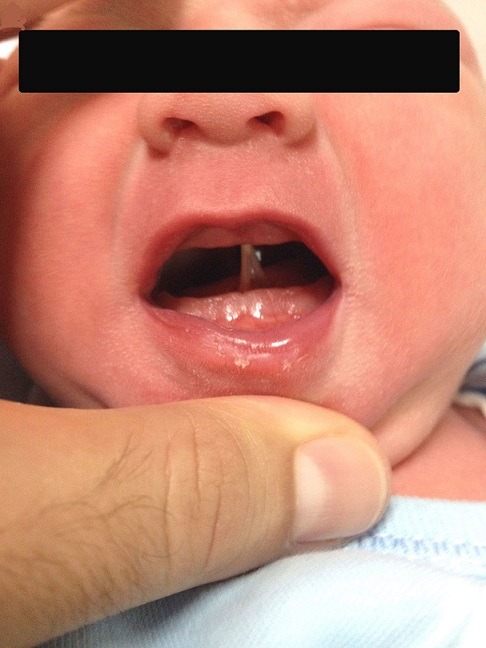
Subglosso-palatal membrane

